# A realistic morpho-anatomical connection strategy for modelling full-scale point-neuron microcircuits

**DOI:** 10.1038/s41598-022-18024-y

**Published:** 2022-08-16

**Authors:** Daniela Gandolfi, Jonathan Mapelli, Sergio Solinas, Robin De Schepper, Alice Geminiani, Claudia Casellato, Egidio D’Angelo, Michele Migliore

**Affiliations:** 1 Department of Brain and Behavioral Sciences, University of Pavia, Pavia, Italy; 2 Department of Biomedical, Metabolic and Neural Sciences, University of Modena and Reggio Emilia, Via Campi 287, 41125 Modena, Italy; 3 Center for Neuroscience and Neurotechnology, University of Modena and Reggio Emilia, Via Campi 287, 41125 Modena, Italy; 4 Department of Biomedical Science, University of Sassari, Sassari, Italy; 5 Institute of Neuroinformatics, University of Zurich and ETH Zurich, Winterthurerstrasse 190, 8057 Zurich, Switzerland; 6 IRCCS Mondino Foundation, Pavia, Italy; 7 Institute of Biophysics, National Research Council, Palermo, Italy

**Keywords:** Neuroscience, Computational neuroscience, Network models

## Abstract

The modeling of extended microcircuits is emerging as an effective tool to simulate the neurophysiological correlates of brain activity and to investigate brain dysfunctions. However, for specific networks, a realistic modeling approach based on the combination of available physiological, morphological and anatomical data is still an open issue. One of the main problems in the generation of realistic networks lies in the strategy adopted to build network connectivity. Here we propose a method to implement a neuronal network at single cell resolution by using the geometrical probability volumes associated with pre- and postsynaptic neurites. This allows us to build a network with plausible connectivity properties without the explicit use of computationally intensive touch detection algorithms using full 3D neuron reconstructions. The method has been benchmarked for the mouse hippocampus CA1 area, and the results show that this approach is able to generate full-scale brain networks at single cell resolution that are in good agreement with experimental findings. This geometric reconstruction of axonal and dendritic occupancy, by effectively reflecting morphological and anatomical constraints, could be integrated into structured simulators generating entire circuits of different brain areas facilitating the simulation of different brain regions with realistic models.

## Introduction

The analysis of neural functions has been promoted by a number of experimental methods^1–3^ that have been progressively tuned to dissect the mechanisms taking part in neurotransmission^4^. In the last decades several computational models have been developed using experimental constraints^5–8^ to fv therefore providing predictive tools to explore pathophysiological conditions^9–11^. However, despite the fact that computational models are becoming effective tools to explore different spatio-temporal scales of neuronal activity in microcircuits^12,13^ the construction of large network models requires a series of approximations, which scale up with network size. Rather than the absolute number of neurons, the bottleneck in this process is determined by the number of connections between neuronal elements required to instantiate realistic connectivity. The connectome^14^ is in fact a key factor in determining the computational capability of circuits and, more generally, in shaping the network input-output^15^.

In this scenario, the advent of High-Performance Computing, coupled with a huge amount of experimental data, boosted the development of extended data-driven spiking neural network models^16–19^. Irrespective of the single cell model employed and the level of electrophysiological detail, the connectivity strategy remains a critical determinant in the construction of networks^20^.

According to the level of biological realism, various connection strategies have been proposed and applied: from randomized connectivity^12^ respecting the proportion of synaptic contacts or convergence/divergence ratios, to sophisticated methods based on anatomical constraints and endowed with connectivity laws^21^ or touch detection algorithms^22^ strictly adhering to morphological constraints. The computational effort required for the process of connecting hundreds of thousands or millions of neuronal elements for the instantiation of these latter strategies must be considered. Variants of “touch detection” algorithms, which have been recently proposed and applied to the generation of neocortical columns^22^, striatal networks^12^ and cerebellar microcircuits^23^ are based on precise morphologies derived from experimental data which are used to estimate the number of connections between neurons. However, despite the use of experimental morphologies to describe extended dendritic and axonal arborization, most brain regions show peculiar anatomical structures (e.g. surface bending or sulci) that strongly limit the possibility to further customize the model through tailored neuronal reorientation^24^. The customization of morphological orientation according to the anatomical constraints of the modelled region would require additional and dedicated computational effort compared to a random neuronal morphological orientation^24^. An alternative connection strategy, based on the generation of spherical probability clouds representing the axonal and dendritic volumes can be adopted to estimate the connectivity between neurons through isotropic or distance-dependent criteria^18,25,26^. An approach based on probability clouds, that has been implemented for the cerebellar network^18^ has been recently combined with morphology-based rules to generate a set of connection rules that can be updated to the available cerebellar datasets^23^. In mixed approaches, probability clouds are integrated with morphological data for neuronal classes^19,27^, attempting to generalize cell-to cell variability, nevertheless the number of available morphologies is usually not comparable with neurons in a real circuit. Neuronal connectivity can be also derived from the conversion of morphological structures into axonal and dendritic mass distribution within a grid of voxels resulting in neuritic density fields whose intersections would determine the probability of contact^28,29^. The major issue in connecting neurons through morphologies is related to the limited availability of datasets, which requires the cloning and random reorientation of individual morphologies, with an overall increase of the computational cost.

Given the number of neurons typically included in full-scale circuits, and the specificity of morpho-anatomical constraints, network models of extended brain regions with the resolution of single cells have been limited to the olfactory bulb^30^ and the striatum, where the majority of neuronal components can be assumed to be isotropically oriented^31^, and a few regions of neocortex^17^, cerebellum^18^ and hippocampal subregions^13,32^ where strong anisotropies can be observed (e.g. cortical columns or cerebellar parallel fibers). Several brain areas show in fact a strong directionality in the connectivity patterns which need to be taken into account when building network model architecture. One typical example is the hippocampus which exhibits a peculiar anisotropic organization of neurons and axons in both position and orientation depending on the relative location of the cell soma within the hippocampal volume. The connectivity appears to be organized in such a way as to generate a preferential direction for the well-known stream of information going from the dentate gyrus to the subiculum, which has been hypothesized to be the basis for ripples and oscillatory activity^33,34^. Although different models of hippocampal neurons have been generated, ranging from detailed biophysically and morphologically accurate models^35^ to advanced point-neuron integrate-and-fire implementations^36^, full-scale models of hippocampal regions, such as DG, CA1, or CA3 areas based on realistic morpho-anatomical connectivity constraints are not yet available.

In the current work, we present a full-scale reconstruction of the mouse CA1 hippocampal region that can be equipped with any point neuron model. In order to do so, we developed and applied a statistical/geometric approach allowing us to customize local connectivity starting from morpho-anatomical constraints obtained from morphological data. The method, which can be applied to other brain regions, resulted in a connectivity consistent with experimental findings on real brain networks at single cell resolution, and supported experimental suggestions on the preferential direction of signal propagation within the CA1 network.

## Materials and methods

**Figure 1:**
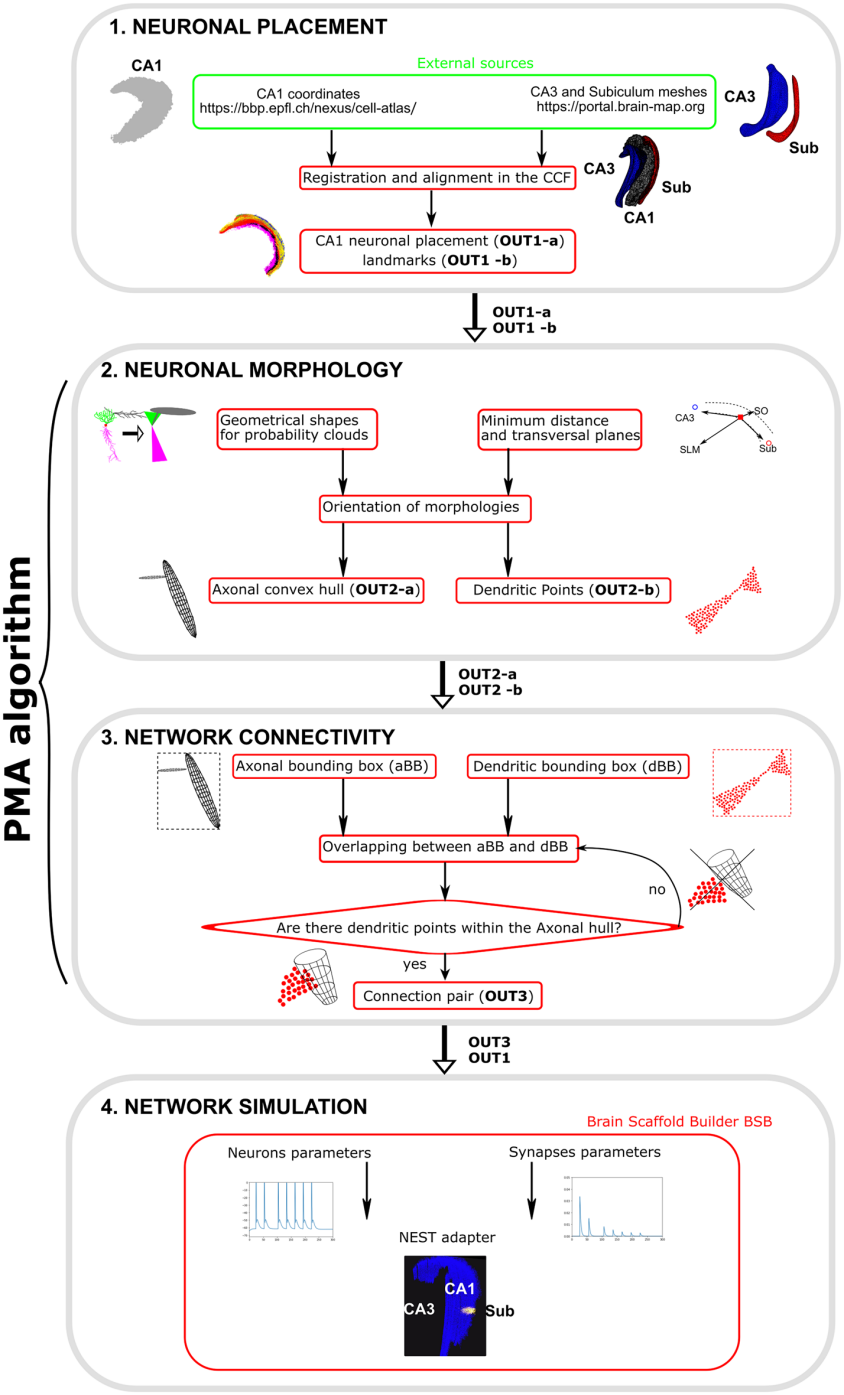
Workflow for the generation of network simulation of CA1 hippocampus. Gray boxes represent the adopted procedural blocks. Red boxes represent computational functions. Green box represents non elaborated data obtained from public repositories (external sources). (1) Neuronal placement: Cell coordinates have been downloaded from the BBP atlas and landmarks have been obtained as meshes from Allen database. 3D coordinates have been assigned to excitatory (2) and inhibitory (11) classes according to their position in CA1 layers (see Fig. 2) and have been aligned with meshes in the CCF to generate the first 2 outputs (OUT1-a,b). (2) Neuronal morphologies: Geometrical shapes have been generated by analyzing morphological databases providing dendritic and axonal features. Neurons have been oriented according to their relative position within the hippocampus and by using CA3, Subiculum and CA1 layers as landmarks to generate axonal convex hulls (OUT2a) and dendritic point clouds (OUT2b). (3) Network connectivity; Bounding boxes for axons and dendrites were generated to calculate the potential intersections of every axon with all dendrites. An iterative algorithm calculated the intersections of every axonal hull only with neurons with overlapping dendritic bounding boxes. Connection pairs (OUT3) were generated when at least one dendritic point was included into the axonal hull. (4) Network simulation. Neuronal placement and connection pairs were loaded into the BSB python module which generated the adapter for the NEST simulator to run the simulation. The PMA algorithm is represented by blocks 2 and 3.

The proposed method has been developed and validated on a full-scale (~ 300 k cells) mouse right hippocampal CA1 circuit following the procedural steps depicted in the flowchart diagram of Fig. 1 and that are explained in detail in the subsequent paragraphs.

### Neuronal placement

**Figure 2:**
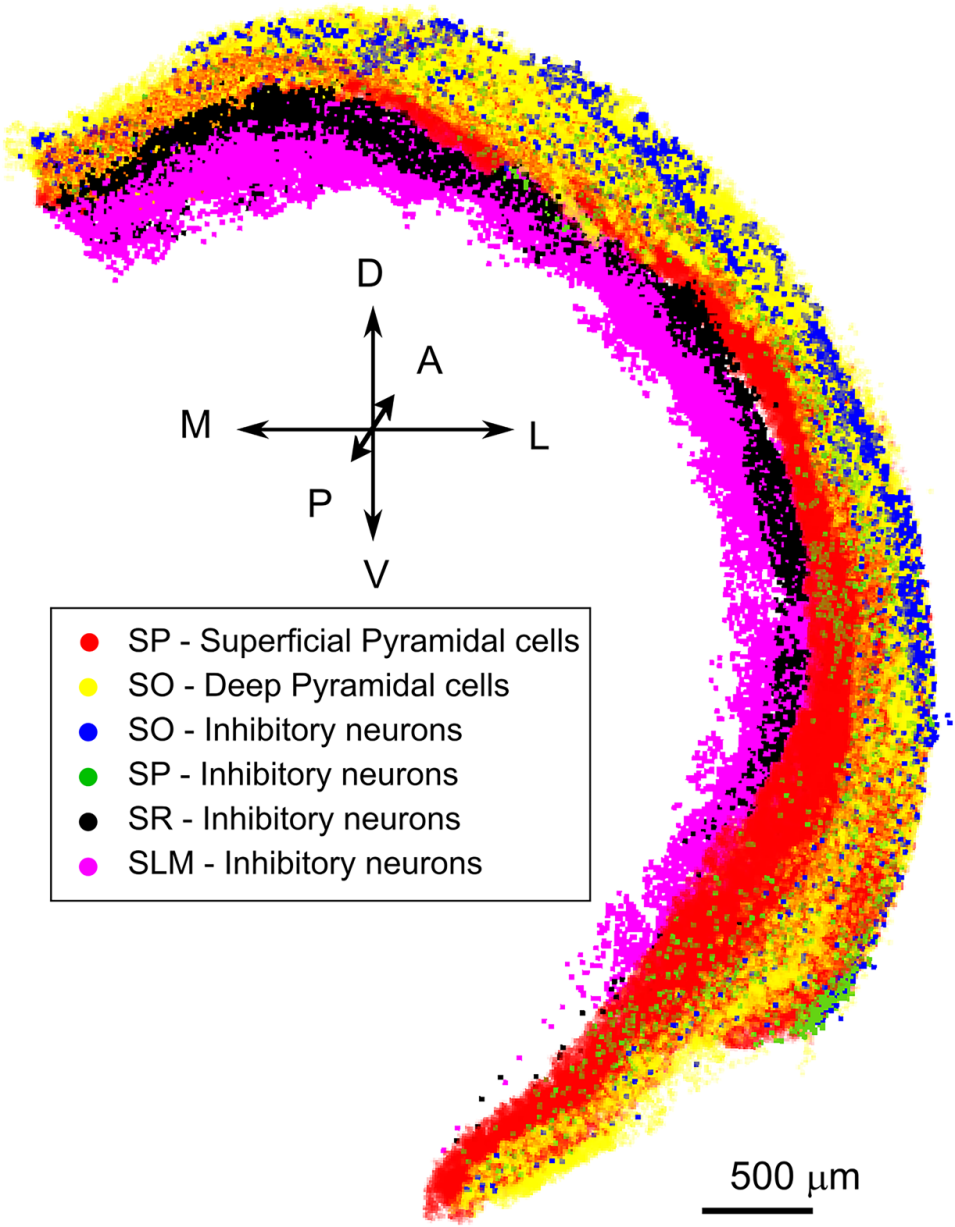
Neuronal placement. The figure shows the excitatory and inhibitory cell placements according to the CA1 region layer subdivision provided by the Blue Brain cell Atlas. Colored dots represent excitatory or inhibitory neurons that were divided according to their positioning within the 4 CA1 layers (SO, SP, SR, SLM): (1) red: Superficial Pyramidal Cells (SP) (2) yellow: Deep Pyramidal Cells (SO) (3) blue: Stratum Oriens Inhibitory neurons (SO) (4) green: Stratum Pyramidalis Inhibitory neurons (SP) (5) black: Stratum Radiatum Inhibitory neurons (SR) (6) magenta: Stratum Lacunosum Inhibitory neurons (SLM).

Cells placement was performed by downloading neuronal coordinates from the Blue Brain Cell Atlas database (https://bbp.epfl.ch/nexus/cell-atlas/)^37^, which provides 3D coordinates of excitatory and inhibitory classes in the Allen reference atlas already annotated for the four layers of mouse hippocampus: Stratum Oriens (SO), Stratum Pyramidalis (SP), Stratum Radiatum (SR) and Stratum Lacunosum Moleculare (SLM) (Fig. 2). Neuronal populations respect the ratio of 10% between inhibitory (inh) and excitatory (exc) classes. The neurons (exc/inh) were then divided into 13 classes (2 exc, 11 inh) according to their relative distribution^38,39^ within layers (see Fig. 2 and Table 1). The scaffolding of the neurons in the simulation volume was performed with the BSB (Brain Scaffold Builder) framework (https://github.com/dbbs-lab/bsb)^23^

**Table 1:** The Table shows the numerical representations of Excitatory Neurons (Deep and Superficial Pyramidal cells) and Inhibitory neurons (Oriens Lacunosum moleculare cells—OLM; Trilaminar cells—Tri, Backprojection cells—BP, cholecystokinin positive Basket cells—CCKBC, Parvalbumin positive cells—PVBC, AxoAxonic Cells—AA, Ivy Cells—IVY, Bistratified cells—BS, Schaffer Collateral associated cells—SCA, Perforant Pathway Associated cell—PPA, Neurogliaform—NG) subdivided on the basis of the placement classes (SO, SP, SR, SLM).

Placement class	Neuronal class	Number
**Excitatory neurons**		
Stratum oriens	Deep pyramidal Cells	45,408
Stratum pyramidals	Superficial pyramidal cells	216,435
**Inhibitory neurons**		
Stratum oriens	OLM, Trilaminar, BP	1979
Stratum pyramidals	CCKBC. PVBC, AA	1661
Stratum radiatum	IVY, BS, SCA, PPA	10,147
Stratum lacunosum molecolare	Neurogliaform	12,397

Since the present method is based on cell positions and on the relative distances between CA1 neurons and targeting subregions (CA3 and Subiculum), we refer to this approach as “*Positional-Morpho-Anatomical*” modeling (PMA). The PMA algorithm, which is detailed in the subsequent paragraphs and in the "Results" section, allows us to generate a full-scale of point neurons starting from cell positions and external landmarks. The CA3 and Subiculum scaffolds required to implement the PMA algorithm have been obtained from the Allen Institute database (http://download.alleninstitute.org/informatics-archive/current-release/mouse_ccf/annotation/ccf_2017/structure_meshes/ply/). The CA1 positioning and external landmarks have been aligned on the Allen Common Coordinate Framework (CCF) and used to generate neuronal morphologies and connections (OUT1a,b in Fig. 1).

### Neuronal morphology

Large-scale neuronal network activity can be simulated by simplifying the description of the electrophysiological properties of the individual neurons. With this aim, point-like neurons, usually modelled as integrate-and-fire^40^ or Izhikevich models^41^, can be adopted to significantly reduce the computational effort^34^. Importantly, the connectivity between cells must be preserved to allow the emergence of the correct functional organization of neuronal activity which in turn requires a specific connectivity rule. In our approach, the rules for the generation of connections between any two neurons were implemented assuming that every neuronal class is characterized by an average shape. Morphological analysis has been performed by collecting the experimentally reconstructed morphologies of CA1 neuron subtypes from the literature and from public databases such as neuromorpho (http://neuromorpho.org/)^42^, Allen Brain Institute^43^ (https://portal.brain-map.org), and Janelia Research Campus^44^ (http://mouselight.janelia.org/). In this analysis, we have assumed that each cell class could be represented as a combination of geometrical shapes (ellipsoids and cones). The parameterization of these shapes (axonal and dendritic extensions) was generated by creating normal distributions for each of the parameters with peaks corresponding to the average values derived from the analysis and half-widths of 10% of the peaks (Table 1-SM). The dimensions of axons and cones for each neuron were then randomly sampled through an automatic procedure within the parameter distributions. The modeling of neuronal morphologies as combinations of ellipsoids and cones mimics the cross-section volume of pre-synaptic axons and post-synaptic dendrites (See Fig. SM-1). The description of the geometrical shapes adopted for each neuronal class is detailed in the "Results" section.

**Figure 3:**
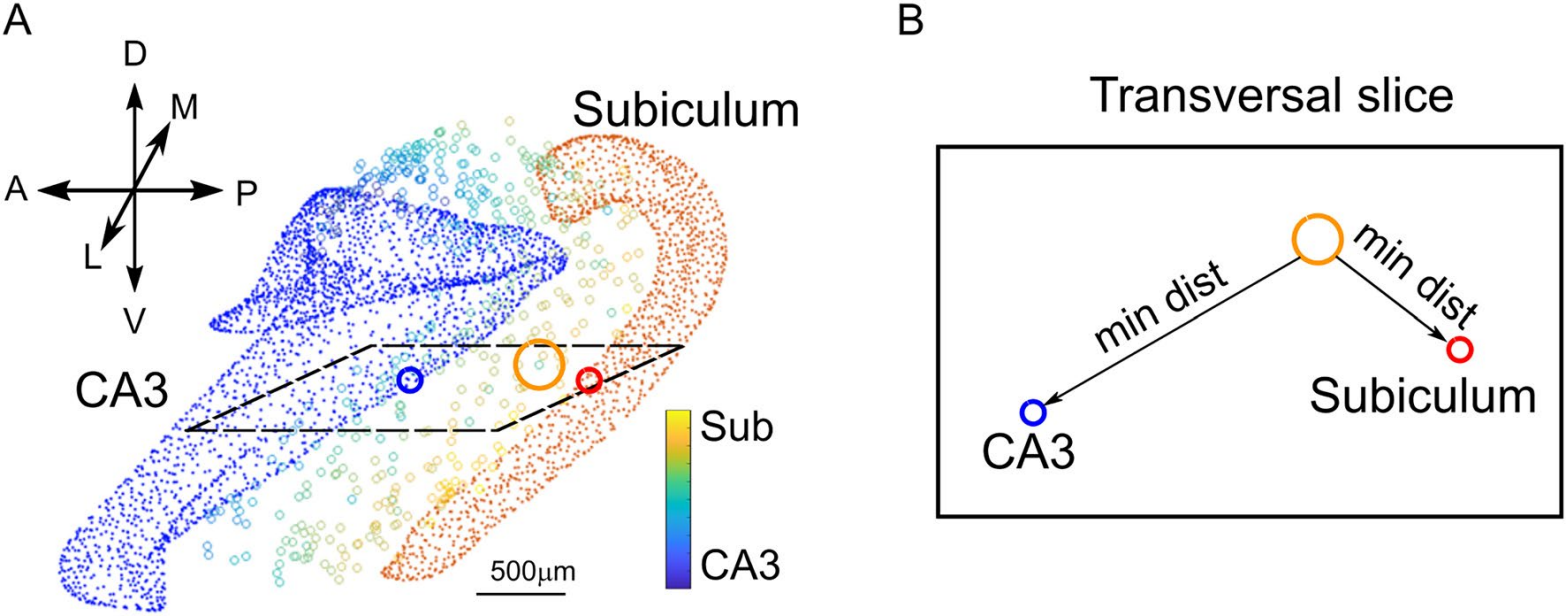
The PMA modeling. (**A**). Relative distance of CA1 PCs (colored circles) from either CA3 (blue dots) or Subiculum mesh (orange dots). Circles are colored according to a distance gradient (see color bar): from blue (PCs are closer to CA3) to yellow (PCS are closer to subiculum). (**B**). The plane formed from the two minimum distance vectors (see A) constitutes the transversal plane adopted to orient the major semiaxes of the axonal branch extent.

In addition to the size of geometrical shapes, neurons were endowed with morpho-anatomical features derived from the calculation of the minimum Euclidean distances of neuronal soma from internal (CA1 layers) and external landmarks (CA3 and Subiculum; see Fig. 3). An automatic iterative algorithm scanned neuronal positions and calculated the minimum distances between neuronal coordinates and CA3, Subiculum meshes and CA1 layers. This allowed the consideration of the experimentally observed preferential orientation of PC axons along the direction of the minimum distance between CA3 and Subiculum, thus implementing the strong directionality in Pyramidal-to-Pyramidal activity propagation along transversal hippocampal slices that has been observed experimentally^45^. These morpho-anatomical features were used to generate the parametric description that allowed the orientation of axonal and dendritic probability clouds along realistic anatomical axes. All the algorithms dedicated to the identification of landmarks and to neuronal morphology modeling were written in Matlab (The Mathworks inc.).

### Ellipsoid

Assuming that any quadratic function f (x_1_, . . . , x_n_) can be written in the form ${X}^{T}QX$, where $Q$ is a symmetric matrix ( ), given a system of eigenvectors (unit vectors) that diagonalize the symmetric matrix, any ellipsoid can be described as a volume oriented in the direction set by the eigenvectors and elongated along the semi-axis as set by the eigenvalues.

The probability ellipsoid representing axonal projections can thus be easily parameterized considering an orthonormal system of eigenvectors **v**_**1,**_** v**_**2,**_** v**_**3**_ associated, respectively, with the eigenvalues λ_1_, λ_2_, λ_3_ of a 3 × 3 symmetric positive matrix M. If.

1 $$ V = \left[ {v_{1,} v_{2,} v_{3,} } \right] $$

Then.

2 $$ V^{T} MV = \left[ {\begin{array}{*{20}c} {\lambda_{1} } & 0 & 0 \\ 0 & {\lambda_{2} } & 0 \\ 0 & 0 & {\lambda_{3} } \\ \end{array} } \right] = D\left[ {\lambda_{1} ,\lambda_{2} ,\lambda_{3} } \right]; $$

is a diagonal matrix containing the eigenvalues of M and the normalized vectors **v**_**1**_, **v**_**2**_, **v**_**3**_ are called the principal axis of M (Fig. SM-1).

By virtue of this property, we obtained (see Eq. 2) the symmetric matrix ($Q$) starting from an arbitrary base of orthonormal vectors **u**_**1**_, **u**_**2**_, **u**_**3**_ (ellipsoid orientation vectors) forming the matrix U and diagonal matrix of arbitrary eigenvalues (semiaxis lengths) $D$

3 $$ Q = UDU^{T} $$

The orientation vectors were selected according to the directionality of the fibers within CA1 thus, the orientations of probability ellipsoids were modelled starting from specific anatomical landmarks. The first constitutive landmark is represented by the relative positioning of each CA1 neuron with respect to other hippocampal regions. The minimum distance vectors between CA1 neurons and CA3 and subiculum structure meshes allowed the construction of transversal planes in correspondence to each cell (Fig. 3).

For instance, in the case of Pyramidal cells the transversal orientation vector (**u**_**1**_) was taken along the direction of the PC-Subiculum minimum distance vector and constituted the ellipsoid major axis orientation. The orientation vector along the longitudinal direction (**u**_**2**_) was taken from the cross product between PC-Subiculum and PC-CA3 minimum distance vectors. The orientation along the vertical direction (**u**_**3**_) was taken as the cross product between **u**_**1**_ and **u**_**2**_. The probability cloud associated with the ellipsoid was then modelled as scattered tridimensional points following the canonical parametric equations:

4 $$ x = \lambda_{1} cos\vartheta sin\varphi ,y = \lambda_{2} cos\vartheta sin\varphi ,z = \lambda_{3} cos\varphi $$

where $$0\le \vartheta \le 2\pi $$ and $$-\pi \le \varphi \le 0$$.

The points composing the ellipsoid probability cloud were obtained by generalizing the canonical parametric equations to an arbitrary orientation based on the aforementioned orientation vectors.

### Cones

Apical and Basal dendritic arborizations have been modelled as conical point cloud volumes with extent and orientation based on morpho-anatomical constraints. To parameterize conical probability clouds, we assumed that **u** and **v** are two orthogonal vectors that lie in the plane of the circle forming the basis of the cone. To build a cone between point $O$ (apex) and base center point (P) with a given radius $R$ we determined the norm of the cone base plane, which is given by $d=P-O$ (Fig. SM-1) The probability cloud associated with the cone was then modelled as scattered tridimensional points following the general parametric.

equation.

5 $$  \left[ {\begin{array}{*{20}c}    x  \\    y  \\    z  \\   \end{array} } \right] = \left( {\begin{array}{*{20}c}    {O_{x}  + \frac{h}{H}dx}  \\    {\begin{array}{*{20}c}    {O_{y}  + \frac{h}{H}dy}  \\    {O_{z}  + \frac{h}{H}dz}  \\   \end{array} }  \\   \end{array} } \right) + \left( {\begin{array}{*{20}c}    {R \cdot \frac{h}{H} \cdot cos\vartheta  \cdot u_{x} }  \\    {R \cdot \frac{h}{H} \cdot cos\vartheta  \cdot u_{y} }  \\    {R \cdot \frac{h}{H} \cdot cos\vartheta  \cdot u_{z} }  \\   \end{array} } \right) + \left( {\begin{array}{*{20}c}    {R \cdot \frac{h}{H} \cdot sin\vartheta  \cdot v_{x} }  \\    {R \cdot \frac{h}{H} \cdot sin\vartheta  \cdot v_{y} }  \\    {R \cdot \frac{h}{H} \cdot sin\vartheta  \cdot v_{z} }  \\   \end{array} } \right)0 \le h \le H,0 \le \vartheta  \le 2\pi   $$

where $$H=\left|P-O\right|=d$$.

Morphological structures were modeled as convex hulls in case of axons or, in case of dendrites as a variable number of scattered points that scaled according to the volume of the dendritic shape. In particular, each point was representative of a volume of about 8000 µm^3^, corresponding to 20 µm side voxel. For example, the apical dendrite of a deep pyramidal cell, that was modeled as a cone (400 µm Height and 80 µm radius, see Table 1-SM), has a total volume of about 2,680,000 µm^3^. The number of points of the apical dendrite of a superficial pyramidal cell was therefore 335, resulting from the ratio between the total dendritic volume and the volume of a single point (2,680,000/8000).

## Network connectivity

Neuronal connectivity was instantiated by iteratively intersecting a single presynaptic axonal hull and the postsynaptic dendritic points of all the neurons belonging to a particular class (Fig. 1-SM). To reduce the computational effort required to explore all the possible connections, we circumscribed axonal and dendritic probability clouds within their minimal bounding-boxes. We first evaluated the intersection between bounding boxes (${BB1}_{3D},{BB2}_{3D}$) by applying the following set of equations to identify overlapping vertices in tridimensional space:

6 $$ BB1_{3D} = \left( {x:(x_{min1} , x_{max1} } \right), y:\left( {y_{min1} ,y_{max1} } \right),z:\left( {z_{min1} ,z_{max1} } \right)) $$

7 $$ BB2_{3D} = \left( {x:(x_{min2} , x_{max2} } \right), y:\left( {y_{min2} ,y_{max2} } \right),z:\left( {z_{min2} ,z_{max2} } \right)) $$

8 $$  \begin{aligned}   overlap3D\left( {BB1_{{3D}} ,BB2_{{3D}} } \right) &  = overlap1D~\left( {BB1_{{3D}} .x,BB2_{{3D}} .x} \right)~\& ~ \\    ~ & \quad overlap1D~\left( {BB1_{{3D}} .y,~BB2_{{3D}} .y} \right)~\& ~ \\     & \quad overlap1D~\left( {BB1_{{3D}} .z,~BB2_{{3D}} .z} \right) \\  \end{aligned}   $$

where, given $BB1_{1D} = \left( {x_{min1} ,x_{max1} } \right)$ and $BB2_{1D} = \left( {x_{min2} ,x_{max2} } \right)$

$$ overlap1D\left( {BB1_{1D} ,BB2_{1D} } \right) = x_{max1} \ge x_{min2} \,\,\&\,\, x_{max2} \ge x_{min1} $$

The final evaluation of the intersection between axonal clouds and dendritic points was performed only on neurons with overlapping bounding boxes (see "Results"). Connection pairs were computed through an iterative algorithm that calculated if at least one postsynaptic dendritic point was included in a presynaptic axonal cloud. All the neuronal pairs that shared at least one point were included in the pool of potential connection pairs. The final number of connection pairs was obtained through a pruning procedure that followed the estimation of the numerosity of connections between two neuronal classes. This number was estimated by multiplying the number of neurons composing the two classes and the synaptic connection probability obtained from hippocampome.org^35,50^. In particular, the connection probability was generated by taking into account synaptic densities and synaptic contacts. For instance, the estimated number of connections between superficial Pyradmidal Cells and Ivy cells can be obtained by multiplying the total number of neurons in the two classes (216,435—PCs and 5074—IVY) per connection probability of the two cell types (0.000933 as obtained from hippocampome.org). The result is 1,024,612 connections. This number has been obtained by pruning the connection pairs generated with the procedure of probability cloud intersection (9,321,511).

The time required to calculate the intersection between probability clouds, including the procedure to generate and orient morphologies, was dependent on the following parameters: (1) the number of points in each cloud (2) the number of potential intersections between each neuron and cells belonging to a specific class and (3) the numerosity of each neuronal class. The first parameter was set a priori and therefore could be adjusted to reduce the computation time whereas the others two parameters depended on morpho-anatomical constraints. Given the numbers adopted to build the CA1 network (see Table 1) the overall procedure terminated on a desktop (32 Gb RAM) in about 36 h with the prevalent time spent on generating the connections among PCs. The full algorithm can be parallelized on a supercomputer to create the full network (~ 300 k neurons and ~ 1 billion synapses) in a couple of hours on 20 CPUs. We have tested the parallel procedure on the Lyra server available at labcsai (http://www.labcsai.unimore.it) which is equipped with an Intel Xeon 20 core 6230 2.1 GHz with 40 processors.

## Network simulation

The proposed method has been developed to generate a connected network of point neurons based on morpho-anatomical constraints, it is therefore not necessarily coupled to specific computational models of neurons and synapses. We have applied the PMA algorithm to the CA1 region of the mouse hippocampus, and we have chosen point neuron and synapse models available in the NEST simulator^46^ (https://www.nest-simulator.org/). It should be noted that this choice has been made to prepare the network to be simulated with short-term plasticity and subthreshold dynamics, however the network simulation can be performed by adopting different point neurons as well as different synaptic computational models. In our case the network simulation has been implemented by providing the neuronal placement and connectivity matrix to the simulation module of the Brain-Scaffold-Builder (BSB) framework^23^. The network configuration implemented to perform the validation tests was composed of “integrate and fire with adaptive threshold” neurons, available in the NEST distribution (“HT_neuron” model^47^). The neurons were connected with “Tsodyks-Markram” synapses, also available in NEST^48^ (“Tsodyks_synapse”). This choice allowed us to simulate realistic short-term synaptic dynamics, using parameters that in most cases were experimentally validated on pairs of connected cells^49^. Among the neuronal models available in NEST, the choice of the “HT_model” was due to the possibility of endowing neurons with “Tsodyks_synapses” on AMPA, NMDA and both types of GABAergic receptors. It should be stressed that our main aim in this case was not to use the present model to predict physiological and pathological activity, but to assess the validity of the procedure to implement the network connectivity and to validate the results against experimental findings. All parameters employed to run network simulations are included in the Supplementary materials. Full scale network test simulations were carried out in parallel on 5 nodes and 36 processors of the Piz-Daint supercomputer available at the Swiss National Supercomputer Center (CSCS, ETH Zurich).

## Results

The approach described in the "Methods" section can be adapted and applied to different brain regions with proper morpho-anatomical constraints. In this section we describe the application of the *“Positional Morpho-Anatomical”* algorithm to the case of the mouse CA1 network, for which data on cell placement and morphological features are available. Several models of hippocampus CA1 have been developed with variable levels of detail ranging from extremely simplified networks^50,51^ to realistic full-scale networks^32^. However, a network at single cell resolution using connectivity rules based on morpho-anatomical constrains, rather than simple fixed connection probabilities, is not yet available. The PMA algorithm is composed of a few procedural steps that in the flowchart diagram (Fig. 1) are included in the boxes `Neuronal Morphology’ and `Neuronal Connectivity’ and that are further detailed in the following sections as: (1) Single cell probability clouds; (2) Probability cloud intersection; (3) Pruning.

## Single cell probability clouds

The different classes of known mouse hippocampal neurons show a high degree of heterogeneity in structural properties. Nonetheless, geometrical constraints based on morpho-anatomical characteristics can be adopted to generate cell morphologies (see "Neuronal Morphology" in "Methods"). By analyzing the morphological features of experimentally reconstructed CA1 neurons obtained from public repositories (see "Methods"), each neuronal subtype was assigned to a unique morphology. All neurons belonging to a specific class were composed of a combination of ellipsoids and cones whose dimensions were randomly chosen within the normal distributions of dendritic and axonal sizes calculated from experimental morphological features (see Table 1-SM).

### Pyramidal cells

**Figure 4:**
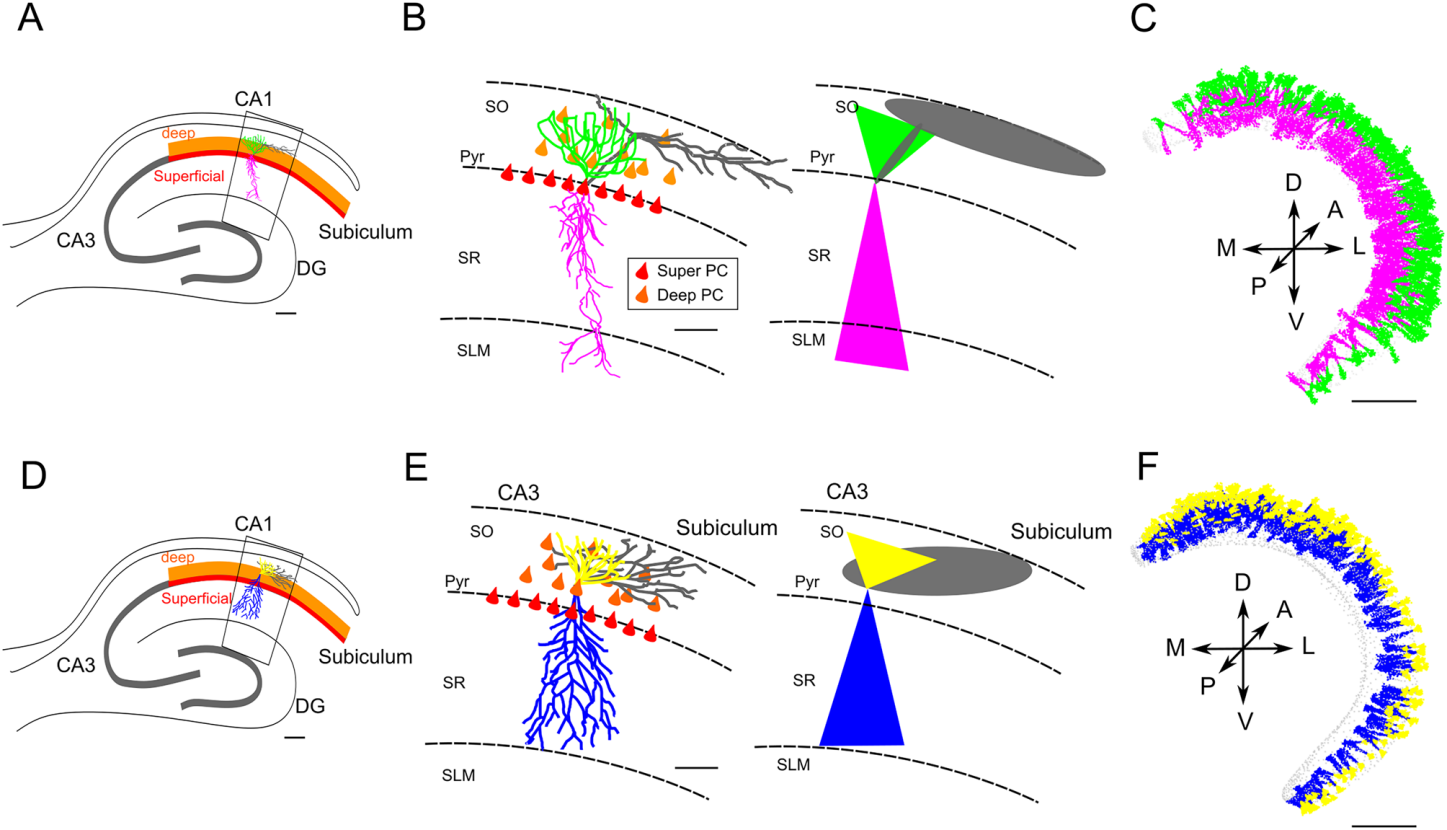
(**A**) Schematic illustration of a transversal section of the hippocampus with major regions. Colored lines illustrate the deep (orange) and superficial (red) subdivisions of the CA1 Pyramidal layers. Inset shows the CA1 region highlighted in panel (**B**). (**B**). left panel; Realistic morphology of a superficial PC (basal dendrites in green, apical in pink, and axon in gray), oriented within a region of a transversal CA1 hippocampal slice (inset of panel **A**). Red triangles correspond to PC soma location within the stratum pyramidalis whereas orange triangles represent the scattered distribution of deep PCs within the SO. right panel; the probability clouds are represented as two triangles (2D of a cone) and an ellipse (2D of an ellipsoid) (color code respects the realistic morphology). (**C**) Reconstruction of dendritic probability clouds of 400 superficial PCs automatically generated and oriented according to cell placement within the CA1 volume. (**D**) Schematic illustrating transversal section of the hippocampus with major regions. Colored lines illustrate the deep (orange) and superficial (red) subdivisions of the CA1 Pyramidal layers. Inset shows the CA1 region highlighted in panel (**E**). (**E**). Similarly to B, a realistic morphology of a deep PC (basal dendrites in yellow, apical in blue, and axon in gray) oriented within a region of a transversal CA1 hippocampal slice (inset of panel **D**). The corresponding probability clouds are represented on the right respecting the color code. (**F**) Reconstruction of dendritic probability clouds of 400 deep PCs automatically generated and oriented according to cell placement within the CA1 volume. Scale bars in µm (40 in **A** and **D**, 100 in **B** and **E**, 1000 in **C** and **F**).

Excitatory pyramidal cells (PCs) were divided into two subpopulations depending on soma positioning: Superficial (SP) and deep (DP) pyramidal cells^52,53^ (Fig. 4). In both cases axons emerging from the cell body project their extensions mainly into the SO contacting dendrites of other pyramidal cells and of GABAergic interneurons. Notably, PCs preferentially orient their axons toward the Subiculum with little divergence to the CA3. Furthermore, axonal arborizations spread in the longitudinal axis of the SO mainly in close proximity to the cell body^54,55^ (Fig. 4). We have therefore created a single ellipsoid to describe the axonal clouds of deep PCs (Fig. 4A–C) since their somas lie in the SO, whereas the axons of superficial PCs were described with a combination of a thin ellipsoid emerging from the soma and projecting to the SO, connected to a larger ellipsoid lying in the SO and pointing preferentially to the subiculum (Fig. 4D–F). The required eigenvectors of the eigenmatrix (see "Methods") were generated from vectors connecting the pyramidal soma coordinates and the minimum distance from subiculum, CA3, SO, SR and SLM.

The probability clouds of each PC, which accounted for the neuronal morphological volume, were automatically oriented in the directions dictated by anatomical constraints. Namely, dendrites were oriented in the directions connecting cell placement with SLM and SO respectively for apical and basal dendrites. Similarly, axonal ellipsoids were directed in the direction connecting cell placement and the subiculum (Fig. 3). This procedure yielded an automatic orientation of all PCs respecting the organization observed experimentally (Fig. 3). Following experimental suggestions^45^, in our model PC axons project unidirectionally towards the Subiculum with a minimum (~ 100 µm) back-projection towards the CA3 and with an overall length proportional to the minimum distance from the subiculum*.*

### Interneurons

**Figure 5:**
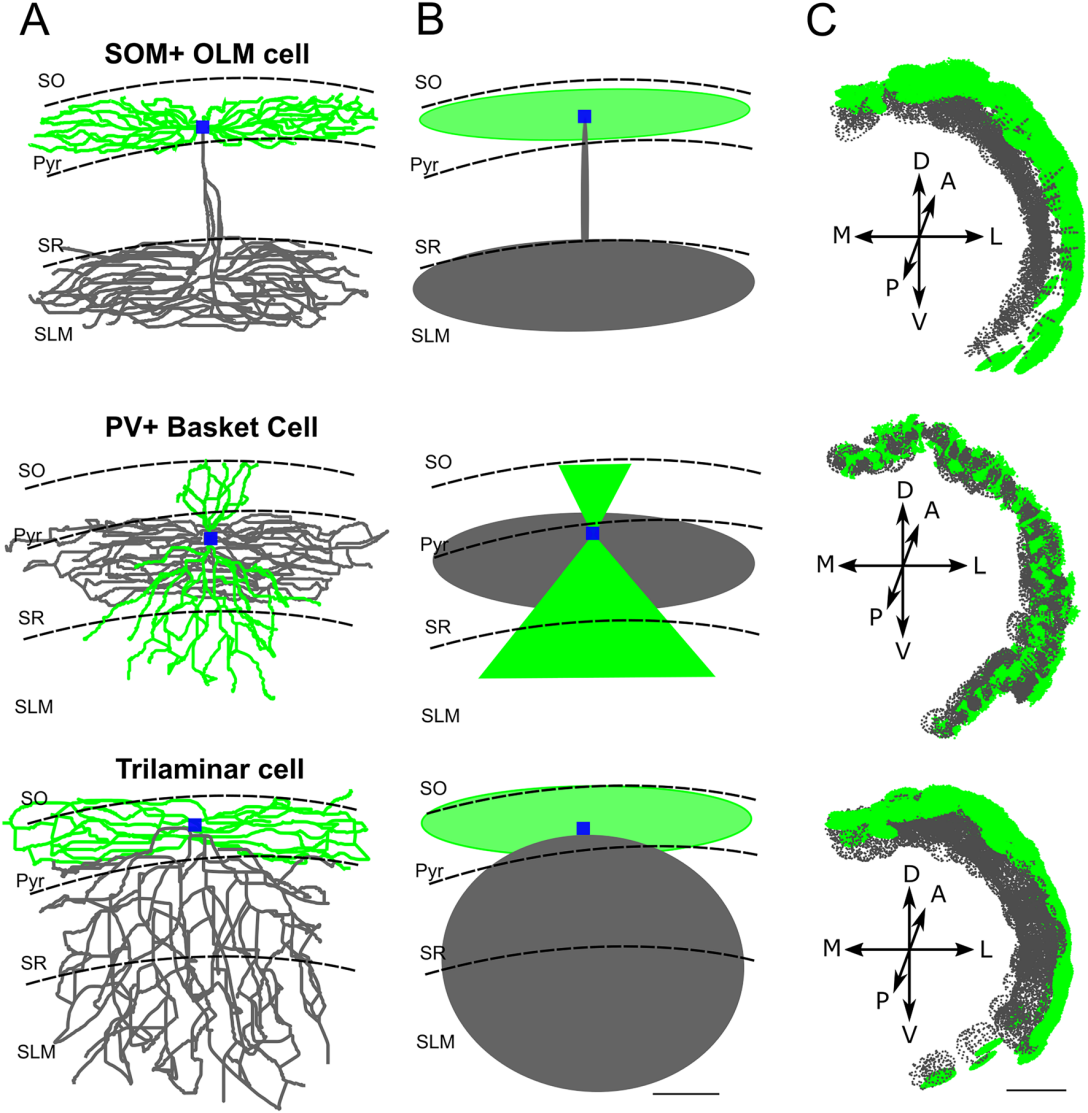
Interneuron morphologies. (**A**) Example of a morphological reconstruction obtained from database neuromorpho.org of three interneurons: Somatostatin positive Oriens interneurons (SOM^+^ OLM); Parvalbumin positive basket cell (PV^+^ Basket cell); Trilaminar cell. Color code is used to represent different morphological compartments (green—dendrites, gray—axons, blue—soma). (**B**) The modelled probability clouds are represented for each of the three neuronal categories with the same color code. (**C**) Probability clouds are positioned in the CA1 respecting the orientation of axons and dendrites with respect to the internal layers and to the external hippocampal subregions (CA3 and subiculum). Scale bar (**A**, **B** 100 µm; **C** 1000 µm).

According to the heterogeneity of shapes and orientations of inhibitory interneurons, we have identified 11 classes of cells which were grouped into 7 different shapes generated through combinations of axonal and dendritic probability:

1. *Perisomatic inhibition.*
  The cell bodies of this class of interneurons, including PV^+^ basket cells, CCK^+^ basket cells and axo-axonic cells, lie mainly in the SP and project a dense axonal cloud within the SP to contact the somas and dendrites^39^ of PCs. Conversely, dendrites travel from SO to SLM crossing transversally the entire CA1. The axon was modeled as a large ellipsoid preferentially extending in the SP, while a pair of cones oriented in the SO-SLM direction and pointing to the cell soma were employed to model apical and basal dendrites (Figs. 4, 5).
2. *SLM projecting neurons*
  This class of interneurons included the SO-OLM and the back-projecting cells and showed the characteristic of projecting a thin axonal filament from the SO where the cell somas are confined, to the SLM where their axons start bifurcating^39^ to give rise to dense axonal plexuses contacting the dendrites of PCs and of other interneurons. Both axons and dendrites were modeled as a combination of ellipsoids whose dimensions and orientations were automatically calculated from morpho-anatomical constraints (Figs. 4, 5).
3. *Concentric axons and dendrites*
  These neurons included Ivy and Bistratified cells whose cell bodies are mainly located in the SR and diffusely project an axonal cloud from SLM to SO isotropically^39^. Similarly, dendrites are preferentially oriented in the direction going from the SO to the SLM and tend to be confined inside the axonal cloud. In our model, axons and dendrites where both designed as single ellipsoids (Fig. 5).
4. *Multilaminar axons*
  Multilaminar axon cells are characterized by an axon starting from the soma in the SO and crossing all CA1 layers^39^. Conversely, dendrites remained closely confined around the soma^36^. In our case, both neurites were modeled as ellipsoids with specific orientation and dimensions given by relative positioning within the CA1 volume (Figs. 4, 5).
5. *Schaffer*
  The Schaffer collateral associated cells are differentiated from multilaminar and concentric interneurons because their soma are predominantly positioned in the SR while their axons and dendrites emerging from the cell body project in opposite directions: the axon projects to the SO while the dendritic arborization projects to the SLM^39^ (Fig. 5). Our axons and dendrites are reproduced as eccentric ellipsoids.
6. *Perforant*
  The Perforant pathway associated cells have their cell bodies in the SR and their axonal extents are confined in the SR and SLM while their dendrites project in both directions^39^. We have modeled the dendrites as two cones with vertex on the soma and a large ellipsoid reproducing the axonal plexus (Fig. 5).
7. *Neurogliaform.*
  The neurogliaform neurons, beside PCs are the most numerous neurons in the CA1. They have somas which are well confined in the SLM together with their short dendrites. A large axon is directed toward the SO with extended projections in the SR^39,56^. In our approach, both axonal and dendritic clouds are modeled as ellipsoids (Fig. 5).

**Figure 6:**
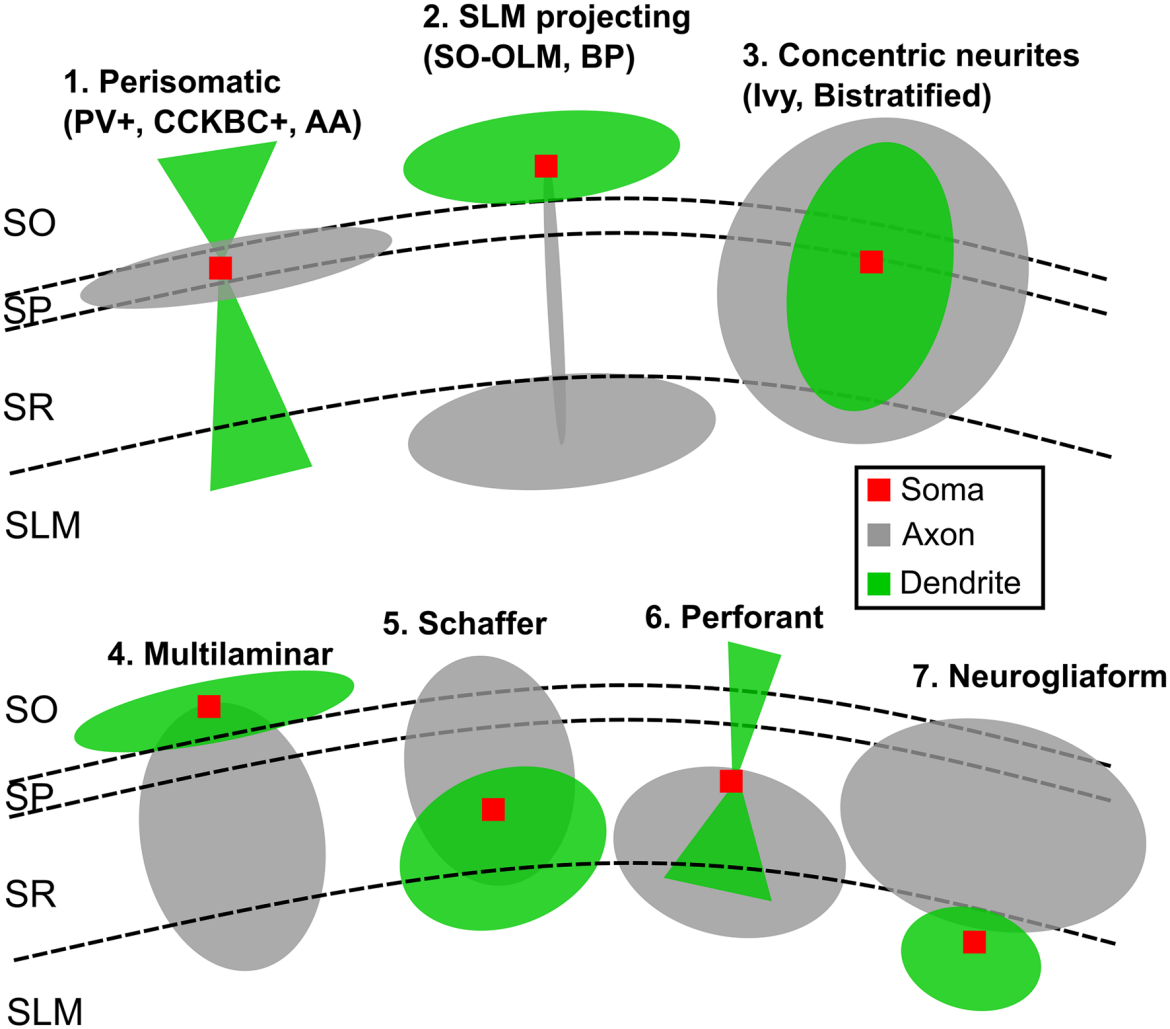
Interneuron morphologies Probability clouds for the classes of morphologies that have been generated to represent the 11 types of interneurons. Red squares indicate the preferential position of the cell soma within the CA1 layers.

The final probability clouds were generated by randomly sampling the size of ellipsoids and cones from a normal distribution of values which was created according to the parameters obtained from the analysis of the experimental morphologies (see "Methods"). This procedure allowed us to generate the entire neuronal dataset (excitatory; Fig. 4 and inhibitory Figs. 5, 6) with different combinations of parameters describing axons and dendrites to account for cell-to-cell variability and to avoid the use of realistic morphologies that need to be replicated and reoriented.

### Probability cloud intersection

The simulation of a point-neuron network requires the determination of which neurons are connected, independently from the number of synaptic contacts. The circuit connectivity has been generated by intersecting presynaptic axonal and postsynaptic dendritic probability clouds for all neuronal classes. Given the extent of axonal and dendritic clouds, it was not necessary to consider the connectivity of each neuron with all the others in the full network. The dimensionality of the intersection space has been reduced using the bounding boxes determined by the cell individual axonal and dendritic probability clouds. The evaluation of the intersection between pre and post-synaptic clouds was therefore performed only on neurons with overlapping bounding boxes (see "Methods"). A customized algorithm tested all the potential connections by assessing the presence of dendritic points of a given postsynaptic neuron within the volume of a presynaptic axonal cloud (Fig. SM-1). In case at least one point was found, the pair of neurons was included into the list of potential connections. The algorithm iteratively scanned all the postsynaptic neurons whose bounding boxes showed a potential overlap with the presynaptic neuron under investigation. The first calculation of the connectome resulting from this procedure generated about 1 billion of connected pairs. However, an estimation of the number of connections in the mouse CA1 of the right hippocampus obtained by intersecting the numerosity of neuronal classes and the connection probability yielded about 120 million connections (see "Methods"). The initial number of synaptic pairs has therefore to be pruned to be consistent with experimental observations.

### Pruning

The experimental quantification of the connected pairs has been performed by considering the probability of connection between two neuronal classes and the total number of neurons of the two classes. The full connectome has therefore been generated by adopting the coupled connection probabilities between all the 13 neuronal classes determined experimentally as in previously published models^38,53^ (see hippocampome.org for the complete list of references). Briefly, a detailed analysis of confocal images allowed the determination of the precise description of neurites, which were then enriched by data on synaptic densities per neuron and interaction distances obtained through electron microscopy. The estimation resulting from this procedure yielded 120 million connections (see "Methods"), which is about one order of magnitude lower than the billion connections resulting from the intersection procedure. We have therefore pruned the connectome by randomly sampling connected pairs to reach the initial estimate for each neuronal synaptic couple. The final connectivity matrix is reported in Table 2SM. The pruning procedure was necessary since the intersection between probability clouds does not consider the effective number of dendritic points intersecting axonal volumes or the density of fibers within volumes and thus overestimates the effective connections.

### Construction validity

**Figure 7:**
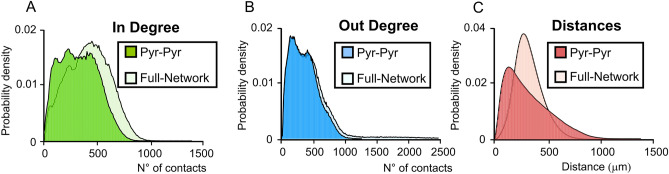
In-degree and Out-degree. (**A**) In-degree distribution shows the number of incoming inputs received by a single neuron expressed as a probability density. The degree distribution for the full network shows that there are about 500 incoming inputs on average. The in-degree distribution for solely excitatory cells is left shifted towards lower values. (**B**) The out-degree distribution represents the number of cells contacted by a single neuron in the network expressed as a probability density. In this case, including inhibitory synapses in the network leads to an enlargement of the curve rather than to a shift, since the peak value is left almost unchanged. (**C**) The connection length distribution shows a shape similar to that determined by experimental and computational studies ^20^.

One of the most common methods adopted to validate a model network is the evaluation of the probability density of converging inputs and diverging outputs, namely the in-degree and out-degree distributions. Network connectivity analysis of different brain regions has shown that the shapes of in-degree and out-degree distributions of neural circuits are well conserved among species. In particular, it has been observed^20^ that in-degree and out-degree distributions can be fit by a convolution between a power law function and an exponential law function^20,57^. This network feature has been observed in the rodent hippocampus (see Fig. 1 in^20^) where neurons with high connectivity have been identified as “*hub neurons*” and play a major role in hippocampal computation^58^. We have therefore calculated the in-degree and out-degree distributions by measuring the number of connections received by each neuron and the number of neurons contacted by each neuron. The resulting in-degree (Fig. 7A) and out-degree (Fig. 7B) probability distributions exhibited a shape consistent with experimental data^59^. This was also true for the distance distribution (Fig. 7C) which was obtained by calculating the number of each given length^20,57,59^.

The in-degree and out-degree distributions of connections among only pyramidal cells showed the expected shapes (Fig. 7A,B) and the shapes were conserved when inhibitory connections were included in the distributions, albeit the model predicts that the peaks of the in-degree distributions shift to higher values, suggesting a prominent role of inhibitory interneurons as hub neurons (Fig. 7A–C).

### Network validation

**Figure 8:**
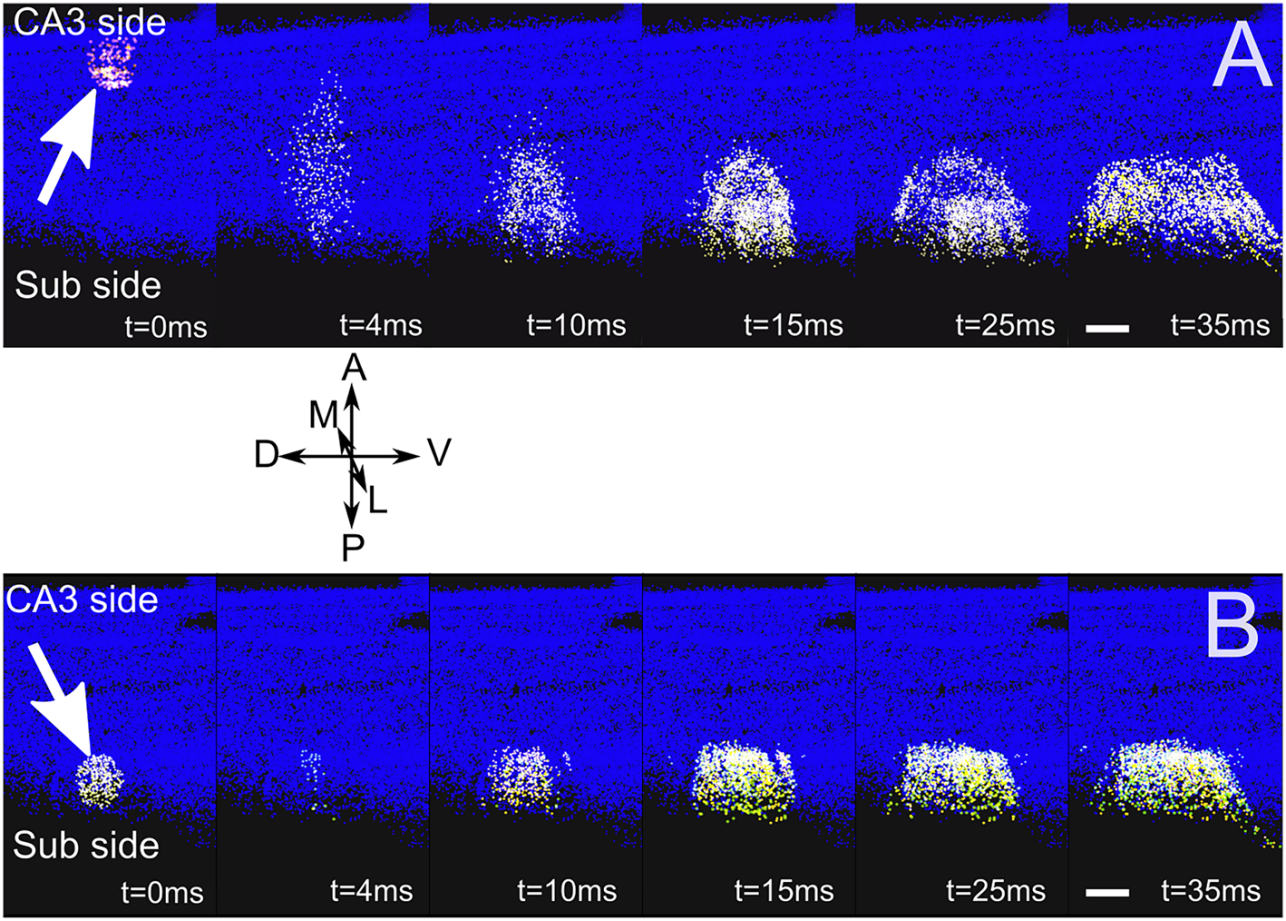
500 µm slice simulation. (**A**). Snapshots from a movie (Suppl. Movie 1) illustrating a simulation of a 500 µm thick transversal slice stimulated from the CA3 side (white arrow). Each dot corresponds to a CA1 neuron/interneuron. The activation of a group of about 200 PCs (white spots on the left) by a single stimulus (t = 0 ms) induces a rapid transversal (Antero-posterior; A-P) invasion of the slice (t = 4,10 ms) which subsequently propagates longitudinally (medio-lateral; M-L). (**B**) Similarly to (**A**), stimulation of the slice from the subiculum side (white arrow). preferentially activates PCs whose activity remains confined to the subiculum side with little longitudinal propagation. Images have been generated through the software Vsimpl^60^. Scale bar 100 µm. A neuron’s activity is color coded from blue (rest) to white (spike), to visualize action potentials, with a fixed 2 ms transition time.

A further step to validate the model has been performed by benchmarking network activity on experimental results obtained from literature, which is a crucial step in testing the model construction. The case of CA1 hippocampus is peculiar since the isolation of the CA1 circuit can be hardly performed through a selective removal of afferent fibers mainly coming from CA3. In most cases, the functional activity of CA1 is detected in thick transversal slices that can be activated through local electrical stimulation on subsets of Pyramidal neurons within the CA1. Unfortunately, the complete isolation of external inputs cannot be achieved. It has been shown that in the absence of synaptic inhibition CA1 activity shows strong directionality from the CA3 side to the subiculum side^42^. We have therefore tested our network by equipping the CA1 network with integrate and fire neurons with adaptive thresholds to model PCs (see "Methods") interacting through excitatory (glutamatergic AMPA and NMDA) synapses exploiting short-term facilitation and depression (see "Methods"). The connectivity methodology has thus been tested by simulating the activation of a portion of the network corresponding to a transverse 500 µm thick slice (Fig. 8).

It has been experimentally shown (see Fig. 1A,C in^45^) that, with the inhibitory contribution blocked, a single stimulus delivered to PCs close to the CA3 side induced firing activity that preferentially propagated along the antero-posterior (transversal) direction. Conversely, when the stimulus was delivered close to the subiculum, the response remained confined without significant backpropagation toward the CA3. In Fig. 8, we show snapshots from a simulation in which the full-scale model was reduced by mimicking a 500 µm thick slice by preserving all connections between pairs of excitatory neurons belonging to the volume of the slice. The stimulation was simulated by recruiting about 200 neurons within a spherical volume with 100 µm radius located either close to the CA3 or on the Subiculum side (See "Methods"). As shown by the frames illustrating the activity during the simulation, the model network nicely reproduced this differential propagation of activity from CA3 to the Subiculum (Fig. 8A), but not vice versa (Fig. 8B).

**Figure 9:**
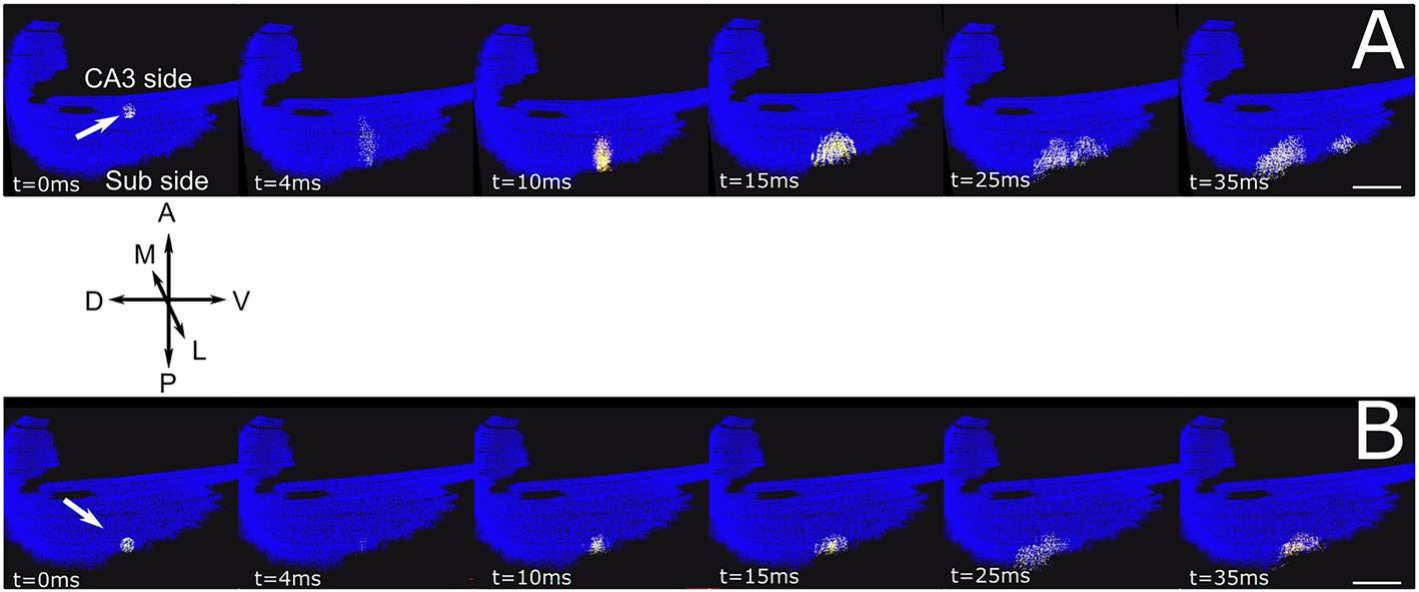
Full-scale network stimulation. Snapshots from a movie (suppl. Movie 2) illustrating a simulation in which the activity was evoked by a single pulse stimulation delivered to about 200 pyramidal cells in a 100 µm diameter sphere near the CA3 side. As in the case of Fig. 8, the activity propagates in the Antero-posterior direction from CA3 to subiculum to subsequently spread longitudinally while remaining confined to the subiculum side. Scale bar 1 mm. As in the case of Fig. 8, a neuron’s activity is color coded from blue (rest) to white (spike), to visualize action potentials, with a fixed 2 ms transition time.

Finally, we have investigated how the model predicted the signal propagation in the full CA1 volume. Also in this case, the model was restricted to excitatory-excitatory connections to reproduce experimental findings shown in^45^ and in^61^. A single pulse delivered either on the CA3 side (Fig. 9A) or on the subiculum side (Fig. 9B) elicited a spread of activity preferentially in the CA3-subiculum direction to subsequently propagate in the longitudinal direction. This observation is in agreement with recent findings obtained with Voltage-Sensitive Dye imaging recordings in whole hippocampus preparation and in longitudinal slices^61^ (Fig. 9).

## Discussion

In this work, we have implemented a bottom-up method to generate a large scale network of point neurons based on morpho-anatomical constraints. It allowed us to reconstruct a full-scale model of the CA1 region of the right murine hippocampal network. The data-driven method implements a geometrical/probabilistic representation of neuronal connectivity fields based on morphological parameters derived from anatomical features of the neurons under investigation. This is particularly valuable in conditions of poor or absent data due to the possibility to generalize putative cell morphologies as abstract geometrical volumes. For instance, by making specific assumptions, it would be possible to generalize the knowledge acquired from rodent experimental data to implement a human hippocampus model. Until recently, random connectivity patterns were widely used to build the connectome of modeled networks^62^ even though this approach does not take into account dendritic or axonal geometries. In recent years, realistic morphologies obtained from experimental reconstructions and converted into digital files have been used to generate network connectivity. Nonetheless, despite the fact that morphological databases are being continuously enriched with new data, the available morphologies are a limited percentage of the neurons required to build full scale networks. This issue is particularly important given the uniqueness of the axo-dendritic trees and their relative orientations in the circuit. The construction pipeline which is used with realistic morphologies typically relies on a few tens of cells which are iteratively cloned to achieve the network size. Four different approaches can be used to clone neurons: (1) randomly changing the neuritic organization, (2) modifying the length of axonal and dendritic branches, (3) randomly reorienting the whole morphologies or (4) generating a synthetic branching respecting morphological constraints.

These procedural steps typically require a remarkable computational cost to run proper algorithms^17^. The connectivity is then generated through microscopic wiring based on rules that respect the dependence of the probability of axo-dendritic contacts on their spatial proximity^13–15,17,63^. This approach is suitable in brain regions where either the morphological organization is anisotropic, allowing the orientation of neurons with a limited number of angles, like the neocortex^15^ and the cerebellar cortex^14^, or quasi isotropic like the striatum^12^ where a random orientation of neuronal morphologies can be accepted. Conversely, this approach is impractical in most brain areas because of the constraints imposed by the morpho-anatomical organization of the network and because of the small sizes of morphological datasets. This is particularly true for the case of the hippocampus, where cells and neurites have polarized orientation with variable angles and the amount of morphologies available is limited.

The PMA method accounts for cell-to-cell variability since each of the 300 k neurons has its own set of morphological parameters coupled with a specific orientation respecting cell positioning within the hippocampal volume. Furthermore, the method can in principle be used to implement, by defining a reference frame for each cell, the polarization of connectivity observed in most brain areas^64,65^. Although the use of probability clouds is not novel in the construction of network connectivity^18,24,25^, the PMA method has the main advantage of accounting for asymmetries in neuronal morphologies that can be mimicked by combining a variable number of ellipsoidal and conical shapes. Moreover, the choice of shaping probability clouds rather than adopting full morphologies allows the unequivocal identification of axes that can be used as reference frames to orient every neuron in the network and differently from models based on touch algorithms and realistic morphologies^63^, our approach allows the separate reorientation of axons and dendrites without geometrical constraints imposed by fixed morphologies. As a consequence, the PMA method can be also implemented in those cases where only a few morphologies, or none, are available, such as in the case of human brain circuits. By adopting this strategy, each neuron’s connection probabilities were kept fixed in all directions, reducing the complexity of calculations and increasing computational performance. Variants of the connectivity algorithm which include the dependence of synaptic probabilities on spatial variables could be envisaged in cases where there is supporting experimental data.

The importance of morphologies in the creation of a connectome has been repeatedly highlighted since the seminal work by Peters and Feldman^66,67^, but has found declining importance in other contexts, such as in the application of graph theory^28^ and predictive algorithms for the creation of network structures^27^. Nevertheless, neuronal morphologies are generally used to create detailed representations of synaptic contacts according to the overlap between neuritic structures. Moreover, the use of realistic morphologies is mandatory for the simulation of biophysically detailed neurons whereas point neuron models simply require the calculation of connection pairs. The proposed method combines the concept of spatial overlap as in the Peters’ rule^66,67^ with a connection probability which is homogeneously distributed within a volume showing a characteristic morphological shape.

Different from other algorithms^21,27^ which are mainly based on a statistical representation of the anatomical space, the present method has the advantage of combining morphological features with the anatomical characteristics of the brain region under investigation. A modeling method^21^ based on the mathematical representations of the anatomical surfaces of each brain region has been recently proposed. This method, which proved extremely efficient in the creation of a whole hippocampus connectome, is based on a series of simplifications: (1) a reduction in the number of neurons and synapses, (2) the neuronal morphology is not considered, (3) the intralayer connectivity is not calculated. Conversely, the proposed PMA algorithm allows the generation of the full connectome of the CA1 region respecting cell positioning, and most importantly is based on the calculation of the total number of connection pairs among neuronal types as derived from literature^38^.

Expanding a previous approach^14^, where the orientation of wiring was performed through distance-based probability functions applied during pruning procedures, the PMA algorithm introduces the orientation of the probability clouds which are used directly to estimate the pairs of connections. With the present connectivity workflow, the randomization of neuronal processes is restricted to the parameter sampling procedure during network construction. It should be noted that while the pruning procedure in the PMA method is, at the moment, based on randomized sampling, in a further development of the algorithm, probabilistic parameterization based on distance could be introduced.

In the last decade, increasing attention has been devoted to elucidating the connectivity matrices of neuronal circuits (connectomics). The development of advanced imaging methods has allowed this issue to be approached experimentally^68^, but a detailed description of the architecture of extended circuits is not yet possible. With the method proposed here, the resulting in-degree and out-degree distributions are consistent with those expected from theoretical and experimental analysis^20,57^. Notably, this result has been obtained without any constraints on the degree distributions, which would be required for the generation of connections through randomized processes.

The connectivity strategy adopted in the present work leads to a CA1 hippocampal network with a strong preferential propagation of activity from the CA3 to the subiculum, which has been suggested to be the basis of the lamellar organization of the hippocampus^69^. Such directionality has been observed in electrophysiological^45^ and imaging recordings^70^ and our approach supports the hypothesis that it derives from the circuit’s connectivity^71^. Although the model network needs to be further validated after fine-tuning the neuronal parameters according to experimental data, the simulation showed functional behaviors observed in slices in terms of both transversal and longitudinal spread^60,72^. This aspect is particularly important since the interlamellar connectivity between excitatory CA1 neurons has been shown to critically regulate the expression of LTP at the CA1-CA1 pyramidal synapses^72^. In conclusion, the proposed method to generate network connectivity appears to be suitable for the creation of connectomes of extended microcircuits showing morphological properties and anatomical constraints that can be synthetized to generate prototype models of axonal and dendritic clouds, opening promising perspectives for the generation of realistic networks of different brain regions.

## Supplementary Information

Supplementary Information 1.

Supplementary Video 1.

Supplementary Video 2.

Supplementary Video 3.

Supplementary Video 4.

## Data Availability

The datasets used to develop and calibrate the algorithm that allowed us to generate the CA1 model are available in the following databases: Blue Brain Cell Atlas (https://bbp.epfl.ch/nexus/cell-atlas/)^37^, neuromorpho (http://neuromorpho.org/)^42^, Allen Brain Institute^43^ (https://portal.brain-map.org), Janelia Research Campus^44^ (http://mouselight.janelia.org/). Furthermore, the PMA algorithm, the full procedure to generate the mouse CA1, and the corresponding data files will be made available as a Matlab toolbox in a dedicated entry on ModelDB (https://modeldb.yale.edu) and in the EBRAINS live papers section (https://ebrains.eu/service/live-papers/).
