# Author Correction: A realistic morpho-anatomical connection strategy for modelling full-scale point-neuron microcircuits

**DOI:** 10.1038/s41598-022-23710-y

**Published:** 2022-11-17

**Authors:** Daniela Gandolfi, Jonathan Mapelli, Sergio Solinas, Robin De Schepper, Alice Geminiani, Claudia Casellato, Egidio D’Angelo, Michele Migliore

**Affiliations:** 1 Department of Brain and Behavioral Sciences, University of Pavia, Pavia, Italy; 2 Department of Biomedical, Metabolic and Neural Sciences, University of Modena and Reggio Emilia, Via Campi 287, 41125 Modena, Italy; 3 Center for Neuroscience and Neurotechnology, University of Modena and Reggio Emilia, Via Campi 287, 41125 Modena, Italy; 4 Department of Biomedical Science, University of Sassari, Sassari, Italy; 5 Institute of Neuroinformatics, University of Zurich and ETH Zurich, Winterthurerstrasse 190, 8057 Zurich, Switzerland; 6 IRCCS Mondino Foundation, Pavia, Italy; 7 Institute of Biophysics, National Research Council, Palermo, Italy

Correction to: *Scientific Reports* 10.1038/s41598-022-18024-y, published online 16 August 2022

The Acknowledgements section in the original version of this Article was incomplete.

“We thank Dr. Adam Ponzi for editorial assistance during the revision phase. This work has received funding from the EU Horizon 2020 Framework Program for Research and Innovation (Specific Grant Agreement 945539, Human Brain Project SGA3) to MM and ED, the Flag ERA JTC 2019 (MILEDI project to MM and SMART-BRAIN project to JM), the “FAR Dipartimentale 2022” to DG.”

now reads:

“We thank Dr. Adam Ponzi for editorial assistance during the revision phase. This work has received funding from the EU Horizon 2020 Framework Program for Research and Innovation (Specific Grant Agreement 945539, Human Brain Project SGA3) to MM and ED, the Flag ERA JTC 2019 (MILEDI project to MM and SMART-BRAIN project to JM), the “FAR Dipartimentale 2022” to DG. MM also acknowledges FENIX computing and storage resources under Specific Grant Agreement No. 800858 (Human Brain Project ICEI), and a grant from the Swiss National Supercomputing Centre (CSCS) under projects ID ich002 and ich011.”

The original Article has been corrected.

