# Supplementary material for: A realistic morpho-anatomical connection strategy for modelling full-scale point-neuron microcircuits
Source: Sci Rep. 2022 Aug 16;12:13864. doi: 10.1038/s41598-022-18024-y (PMC9381785; doi:10.1038/s41598-022-18024-y)
Supplement: Supplementary file 1 — Supplementary Information 1. [file 41598_2022_18024_MOESM1_ESM.pdf]

SUPPLEMENTARY MATERIAL TO THE MANUSCRIPT:

"A realistic morpho-anatomical connection strategy for modelling full-scale point-neuron microcircuits"

Daniela Gandolfi[1,2*†], Jonathan Mapelli [2,3*†], Sergio Solinas[4,5], Robin De Schepper[1], Alice Geminiani[1], Claudia Casellato[1], Egidio D'Angelo[1,6]& Michele Migliore[7].

1 Department of Brain and Behavioral Sciences, University of Pavia, Pavia, Italy
2 Department of Biomedical, Metabolic and Neural Sciences, University of Modena and Reggio Emilia, Via Campi 287, 41125 Modena, Italy.
3 Center for Neuroscience and Neurotechnology, University of Modena and Reggio Emilia, Via Campi 287, 41125 Modena, Italy.
4 Department of Biomedical Science, University of Sassari, Sassari, Italy.
5 Institute of Neuroinformatics, University of Zurich and ETH Zurich, Winterthurerstrasse 190, 8057, Zurich, Switzerland.
6 IRCCS Mondino Foundation, Pavia, Italy.
7 Institute of Biophysics, National Research Council, Palermo, Italy

***** Corresponding authors
[†] Authors equally contributed

Figures

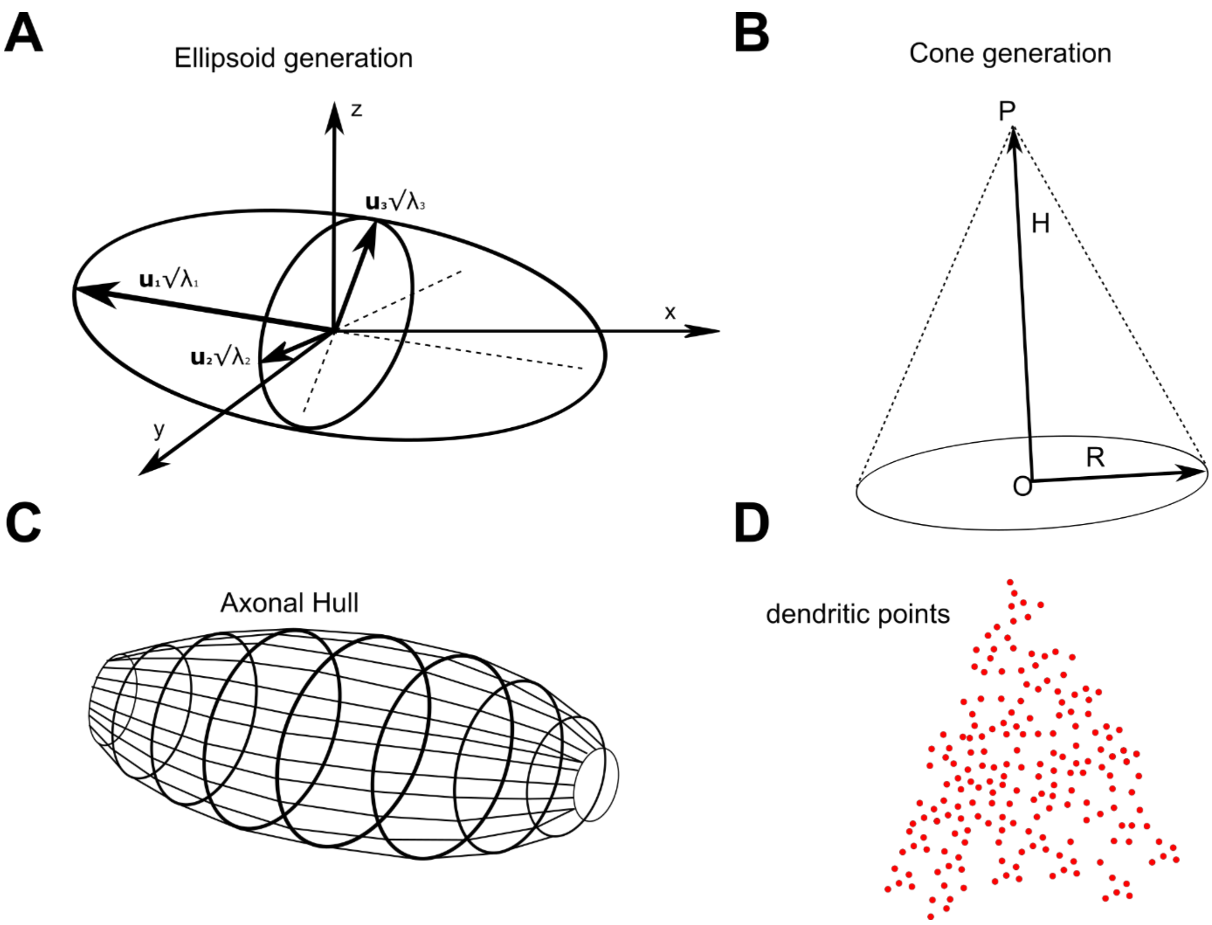

Figure 1-SM. Axonal and dendritic generation. **A**. An ellipsoid is generated through the canonical equation (eq 4 in main text) starting from a base of orthonormal vectors ($\mathbf{u}_1$, $\mathbf{u}_2$, $\mathbf{u}_3$) and a diagonal matrix of eigen values corresponding to the semiaxes of the ellipsoid. Vectors can be oriented according to the reference frame given by hippocampal volume. **B**. Cones are generated throughnthe canonical equation (eq 5 see main text) according the required parametric extension (Height and radius of the base). **C**. Axonal probability clouds are created by converting geometrical shapes in convex hull. **D.** Dendritic clouds are generated by filling the geometric shape with randomly positioned points.

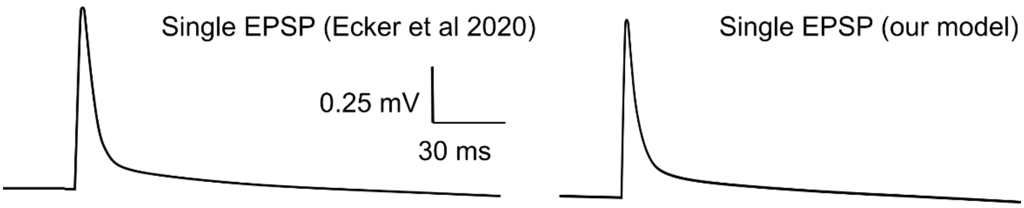

Figure 2-SM. Synaptic model calibration. *Left*. Single EPSP generated in a PC from the activation of a connected PC in a biophysically detailed model of PCs connected qwith excitatory glutamatergic synapse (AMPA and NMDA) as shown in Ecker et al 2020. *Right.* The same single stimulus has been generated in our Integrate and Fire model of PCs activate by an action potential generated by another PC connected with glutamatergic (AMPA and NDMA) synapses modeled with parameters shown in Tables SM-3,4. As it can be evidenced from the comparison of the two traces, the amplitude and the kinetics of the EPSPs are compatible.

NEURONAL CLASS	Axon shape	Axon size (μm)	Dendrite shape	Dendrite size (μm)
Superficial Pyramidal cell	Double Ellipsoid	Axis 1 (150±15)	Cone (apical)	Height (120±12)
		Axis 2 (50±5)		
		Axis 3 (proportional to soma positioning)		Radius (70±7)
		Axis 1 (10±1)	Cone (basal)	Height (400±40)
		Axis 2 (10±1)		Radius (80±8)
		Axis 3 (proportional to soma positioning)		
Deep Pyramidal cell	Single Ellipsoid	Axis 1 (150±15)	Cone (apical)	Height (150±15)
		Axis 2 (50±5)		Radius (75±8)
		Axis 3 (proportional to soma positioning)	Cone (basal)	Height (500±50)
				Radius (90±9)
PVBC	Ellipsoid	Axis 1 (220±22)	Cone (apical)	Height (150±15)
		Axis 2 (220±22)		Radius (130±13)
		Axis 2 (70±7)	Cone (basal)	Height (Proportional to the distance from SLM)
				Radius (70±7)
OLM	Ellipsoid (projection)	Axis 1 (10±1)	Ellipsoid	Axis 1 (300±30)
		Axis 2 (10±1)		
		Axis 3 (Proportional to the distance from SLM)		Axis 2 (300±30)
	Ellipsoid (plexus)	Axis 1 (300±30)		
		Axis 2 (300±30)		Axis 3 (120±12)
		Axis 3 (70±7)		
IVY	Ellipsoid	Axis 1 (220±22)	Ellipsoid	Axis 1 (120±12)
		Axis 2 (220±22)		Axis 2 (120±12)
		Axis 3 (proportional to Distance from SLM and SR)		Axis 3 (proportional to Distance from SLM and SR)
TRI	Ellipsoid	Axis 1 (250±25)	Ellipsoid	Axis 1 (250±25)
		Axis 2 (250±25)		Axis 2 (250±25)
		Axis 3 (Proportional to distance from SLM)		Axis 3 (50±5)

SCA	Ellipsoid	Axis 1 (200±20)	Ellipsoid	Axis 1 (130±13)
		Axis 2 (200±20)		Axis 2 (130±13)
		Axis 3 (Proportional to distance from SLM and SR)		Axis 3 (Proportional to distance from SLM and SR)
PPA	Ellipsoid	Axis 1 (250±25)	Cone (apical)	Height (Proportional to distance from SLM)
		Axis 2 (250±25)		Radius (100±10)
		Axis 3 (Proportional to distance from SLM)	Cone (basal)	Height (Proportional to distance from SO)
				Radius (80±80)
NGF	Ellipsoid	Axis 1 (100±10)	Ellipsoid	Axis 1 (350±35)
		Axis 2 (100±10)		Axis 2 (250±25)
		Axis 3 (100±10)		Variable according to soma positioning
CCKBC	Ellipsoid	Axis 1 (250±25)	Cone (apical)	Height (Proportional to distance from SLM)
		Axis 2 (250±25)		Radius (100±10)
		Axis 3 (70±7)	Cone (basal)	Height (250±25)
				Radius (80±8)
BS	Ellipsoid	Axis 1 (220±22)	Ellipsoid	Axis 1 (120±12)
		Axis 2 (220±22)		Axis 2 (120±12)
		Axis 3 (proportional to Distance from SLM and SR)		Axis 3 (proportional to Distance from SLM and SR)
BP	Ellipsoid (projection)	Axis 1 (10±1)	Ellipsoid	Axis 1 (275±28)
		Axis 2 (10±1)		
		Axis 3 (Proportional to the distance from SLM)		Axis 2 (275±28)
	Ellipsoid (plexus)	Axis 1 (200±20)		
		Axis 2 (200±20)		Axis 3 (80±8)
		Axis 2 (75±8)		

AA	Ellipsoid	Axis 1 (250±25)	Cone (apical)	Height (Proportional to distance from SLM)
		Axis 2 (250±25)		Radius (70±7)
		Axis 3 (70±7)	Cone (basal)	Height (150±15)
				Radius (130±13)

Table 1-SM. Probability clouds parametrization. Parameters adopted to generate ellipsoidal and conical probability clouds for all the neurons in the CA1 network. Estimates are provided in μm.

CONNECTIVITY MATRIX		PRE												
		PYRS	PYRD	TRI	SCA	PVBC	PPA	OLM	NGF	IVY	CCKBC	BS	BP	AA
POST	PYRS	59585707	12501064	1673882	1954084	838880	2172815	533569	3863728	6457364	826721	3426222	264196	800593
	PYRD	12501064	2622720	351180	409966	175997	455856	111943	810609	1354753	173446	718820	55428	167964
	TRI	279880	58719	1430	1255	0	0	1635	14470	10669	0	3971	228	0
	SCA	242251	50824	11473	1611	10857	9259	3104	27019	40899	3653	18909	751	0
	PVBC	147232	30889	4450	909	5217	3858	1146	9680	17324	2279	8175	672	0
	PPA	288666	60562	2564	2499	8043	12511	4118	36170	64136	4105	8996	776	0
	OLM	375753	78833	2286	4418	0	0	165	0	16443	0	6114	366	0
	NGF	0	0	72616	35312	0	123455	31901	269104	342000	0	143499	3673	0
	IVY	1024612	214963	39362	4240	39580	35658	10790	0	148345	15336	64818	4298	0
	CCKBC	127317	26711	4614	856	5474	4906	1420	12186	17897	2408	8195	712	0
	BS	472961	99227	16391	3283	17238	14259	4282	0	65343	6690	29880	1841	0
	BP	26420	5543	513	1316	0	0	13	0	2259	0	681	60	0
	AA	126926	26629	4305	727	5070	5011	1435	12311	15662	2221	7499	614	0

Table 2-SM. CA1 connectivity matrix. Total number of connections between each neuronal class.

ht_params	values
V_m	-70
tau_m	5
t_ref	2
tau_spike	1.5
theta	-50
tau_rise_AMPA	0.2
tau_decay_AMPA	3
tau_rise_NMDA	9
tau_decay_NMDA	148
tau_rise_GABA_A	1
tau_decay_GABA_A	5

tau_rise_GABA_B	30
tau_decay_GABA_B	250
g_peak_AMPA	1
g_peak_NMDA	1.22
g_peak_h	0
g_peak_NaP	0

Table 3-SM. Neuronal model parameters. The table summarizes the parameters of the HT_neuron used to model PCs and adopted to run the simulation of network validation.

	tsodyks_synapse AMPA	tsodyks_synapse NMDA
receptor_type	1	2
tau_fac	17	17
weight	0.6	0.72
delay	2	2
tau_psc	5	5
tau_rec	671	671
U	0.5	0.5
u	0.5	0.5
x	1	1
y	0	0

Table 4-SM. Synaptic model parameters. The table summarizes the parameters of the Tsodyks_2 synapse used to model both AMPA and NMDA component of glutamatergic connections between PC cells and adopted to run the simulation of network validation.

Suppl. Movie 1A. Simulation of CA1 slice stimulated from CA3 side. The movie shows the activation of CA1 PCs within a 500 μm large slice stimulated by a single stimulus delivered within a sphere containing about 200 PCs in proximity to the CA3 side of the CA1. Cells within the stimulated volume respond with a single action potential which is synaptically propagated to the subiculum side of the slice.

Suppl. Movie 1B. Simulation of CA1 slice stimulated from subiculum side. The movie shows the activation of CA1 PCs within a 500 μm large slice stimulated by a single stimulus delivered within a sphere containing about 200 PCs in proximity to the subiculum side of the CA1. Cells within the stimulated volume respond with a single action potential which is poorly propagated to the CA3 side of the slice, whereas signals tend to propagate longitudinally through the border of the slice.

Suppl. Movie 2A. Simulation of the whole CA1 stimulated from CA3 side. The movie shows the activation of CA1 PCs stimulated by a single stimulus delivered within a sphere

containing about 200 PCs in proximity to the CA3 side of the CA1. Cells within the stimulated volume respond with a single action potential which is rapidly propagated to the subiculum via synaptic activation. The activation spread longitudinally remaining confined to the subiculum border.

Suppl. Movie 2B. Simulation of the whole CA1 stimulated from subiculum side. The movie shows the activation of CA1 PCs stimulated by a single stimulus delivered within a sphere containing about 200 PCs in proximity to the subiculum side of the CA1. Cells within the stimulated volume respond with a single action potential which is poorly propagated to the CA3 side. The activation remains confined to the subiculum border and spread longitudinally.
